# A Qualitative Evaluation of Caregiver Support Services Offered at the Atlanta Veterans Affairs Health Care System

**DOI:** 10.5888/pcd16.180156

**Published:** 2019-02-28

**Authors:** Shannon Biello, Anne Tomolo, Corrine Abraham, Cam Escoffery, Delia Lang, Charlotte Sawyer, Nancy Thompson

**Affiliations:** 1 Rollins School of Public Health, Emory University, Atlanta, Georgia; 2 Atlanta Veterans Affairs Health Care System, Atlanta, Georgia; 3 Emory University School of Medicine, Atlanta, Georgia; 4 Nell Hodgson Woodruff School of Nursing, Emory University, Atlanta, Georgia

## Abstract

**Introduction:**

The Department of Veterans Affairs (VA) provides health care to approximately 300,000 patients with dementia. Recognizing the critical role caregivers play in veterans’ health, the Cognitive Disorders Specialty Care Education Center of Excellence (COE) at the Atlanta VA Health Care System implemented a suite of caregiver support services, including formal programs and resource linkages. We evaluated the effectiveness of these services and identified caregiver-perceived gaps in them.

**Methods:**

We conducted 11 semistructured interviews from November 2016 through February 2017 with caregivers of veterans seen in the COE who had participated in support services. After coding transcripts, we established a codebook of 9 major themes and conducted a thematic analysis of all transcripts.

**Results:**

Caregivers spoke positively of COE caregiver services that offered information on dementia, social support, an emphasis on caregiver well-being and self-efficacy, and methods for behavioral change. Gaps identified included the need for additional dementia information and practical support in such matters as advanced directives and eligibility for VA benefits.

**Conclusion:**

Our findings will inform future improvements to COE caregiver support services, such as an expansion of COE’s caregiver educational content and capacity building of existing components such as resource referrals. These results also highlight opportunities for COE to interface with internal and external organizations to enhance existing caregiver services.

**Summary**

**What is already known about the topic?**

Dementia affects a significant number of elderly US adults. Informal caregivers play a critical role in dementia care, offering uncompensated support to patients outside formal medical settings. To support these caregivers, research teams and agencies are developing evidence-based programs.

**What is added by this report?**

The Atlanta Veterans Affairs Health Care System implemented multicomponent caregiver support services, which included elements such as psychoeducational programs and resource referrals. We demonstrated how these services improved participants’ daily caregiver experiences and identified caregiver-perceived program gaps.

**What are the implications for public health practice?**

By evaluating the utility of these services, we can enhance existing programs and inform other caregiver support strategies.

## Introduction

Dementia affects approximately 14% of US adults aged 70 or older. Prevalence and related health care costs are expected to rise as the overall population ages (1–3), and recent estimates suggest that the annual cost of dementia care may double to $109 billion by 2040 (1). Informal caregivers play a critical role in the US health system, acting as supplemental, uncompensated support outside of clinical spheres for patients with dementia (4). Previous studies identified various consequences of extended caregiving, including psychological distress and poor physical health (5–7). These consequences are related to perceived caregiver burden (8). However, only about 25% of caregivers report use of support services, which could reduce this burden (5).

Research teams and government agencies have implemented, evaluated, and translated evidence-based interventions focused on the needs of dementia caregivers; some programs demonstrated success in reducing caregiver burden and improving caregiver well-being while remaining cost-effective (9–12). The Cognitive Disorders Specialty Care Education Center of Excellence (COE), an interprofessional collaborative practice at the Atlanta Veterans Affairs Health Care System (VAHCS), piloted a multicomponent intervention for primary caregivers of its dementia patients that includes services such as visits with staff social workers, resource referrals, and an evidence-based psychoeducational program, the Savvy Caregiver (13).

The primary objective of our formative evaluation was to examine the effectiveness of COE caregiver support services and referrals by using the Transactional Model of Stress and Coping (TMSC) as a framework (14). A secondary objective was to identify any gaps in these caregiver support services that, once addressed, would improve caregiver experiences as well as COE operations overall.

## Methods

We conducted a qualitative, formative evaluation of COE caregiver support services from August 2016 through May 2017 in partnership with COE leadership, clinic staff members, and Emory University. The COE caregiver intervention components evaluated were the Savvy Caregiver Program, a 5-to-6-session psychoeducational training aimed at preparing dementia caregivers for the challenges of neurocognitive decline (13); the Tele-Savvy Program, a tablet- and internet-based remote adaptation of the Savvy Caregiver Program (15,16); caregiver visits and telephone calls with COE staff members (eg, nurse, social workers); and referrals to internal or external resources.

We used a purposive, nonprobability, convenience sample. The COE staff provided recommendations of eligible participants on the basis of the following criteria: 1) the caregiver was significantly involved in the outside support of a COE clinic patient diagnosed with dementia (ie, primary caregiver) and 2) the caregiver had participated in one or more COE caregiver support services. This evaluation was deemed nonresearch quality improvement by the Atlanta VAHCS Research Office and as such, did not require additional review by the Emory University institutional review board.

One researcher (S.B.) conducted 11 in-depth, semistructured interviews from November 2016 through February 2017 in private VAHCS offices, or by telephone when a caregiver was unable to travel. The participation rate was 65% (11 of 17). In general, nonparticipation was due to travel or caregiver time restrictions. With participant consent, the researcher used a VAHCS-approved device to audio-record all interviews and transcribed these interviews verbatim. Participants did not receive incentives for their time.

We used a standard interview guide developed in conjunction with COE leadership and Emory University faculty. Constructs from the TMSC (eg, primary and secondary appraisals, coping efforts) informed the development of interview questions. We categorized interview questions by the following domains of interest: demographics and other background information, previous caregiving experiences, present caregiving experiences, individual coping strategies, experiences with COE caregiver support services, and perceived gaps in these support services.

We completed transcript coding and analysis using MAXQDA 12 Base software (VERBI GmbH). We used a modified grounded theory approach in which the first collection of interviews informed later data collection and analysis strategies (17). One researcher (S.B.) coded an initial batch of transcripts and worked with the project team to develop and refine a codebook of 9 major themes. She then used the final codebook to code the remainder of the transcripts. A second coder (C.S.) coded 20% of the data to assess intercoder reliability and worked with S.B. to review and discuss all discrepancies between the coded transcripts until consensus was reached. S.B. applied any agreed-upon changes to the remaining transcripts. We synthesized and compared themes across all interviews to generate findings.

## Results

All 11 caregivers were female spouses of veteran patients, with a mean age of 67 years (standard deviation [SD], 4.6 years; range, 57–72 years). Caregivers had been providing support to their spouse for a median of 6 years (range, 1.5–24.0 y) (Table 1).

**Table 1:** Demgraphic Characteristics, Caregivers (N = 11) Participating in Caregiver Support Services, Atlanta Veterans Affairs Health Care System, Atlanta, Georgia, August 2016–May 2017^a^

Characteristic	Value
**Age, mean (standard deviation) [range], y**	67.3 (4.6) [57–72]
**Length of time caregiving, median (range), y**	6.0 (1.5–24.0)
**Female sex**	11
**Relationship to veteran is spouse**	11
**Race**	
White	7
Black	4
**Education**	
Some high school	1
High school graduate	3
Some college	4
College graduate	1
Advanced degree	2
**Lives with veteran**	
Yes	10
No	1

a Values are number unless otherwise indicated.

Caregivers remarked on the VA COE caregiver support services (Table 2). Overall, opinions of COE caregiver support services were positive. Five major themes emerged as effective components of these services: information about dementia, social support, a focus on caregiver self-efficacy, application of behavioral strategies, and an emphasis on caregiver well-being.

**Table 2:** Themes and Sample Remarks from Caregiver Participants (N = 11), Study of Effectiveness of Caregiver Support Services, Atlanta Veterans Affairs Health Care System, Atlanta, Georgia, August 2016–May 2017^a^

Theme	Remark (Participant Identifier)
**Effectiveness of the Cognitive Disorders Specialty Care Education Center of Excellence caregiver services**	
Information about dementia	It had a lot of good information in it. It made me aware of . . . what stage he was in, you know, and what we’ve already been through, and what’s normal, what’s not normal. And what to look for as we go on, you know. . . . I think it really helped me. It gave a lot of good advice. Reading materials and all helped. (P03)
	So they [staff in the caregiver program] have kind of helped me to know that it’s not my fault that this is happening. It’s nothing I can do about it. It’s nothing that I did wrong. It happens, and we have no control over the disease. (P06)
Social support	[T]hey [other caregivers] may have experienced something that I have not done yet. Or my experience may have, you know, if I had shared about it, maybe something they’re not going through yet. And it just kind of gives you a, a heads up on what may or may not be coming. You know, what to look for. (P11)
Caregiver self-efficacy	Well, I don’t feel as overwhelmed. . . . I don’t get as anxious as I used to cause now what I try to do is, first do the things that I know that I can do. If it’s something that I know that I can’t do, then I don’t get overwhelmed about it, you know. I try to find an easier way to get it done. . . . I try to handle one stress at a time. (P09)
Application of behavioral strategies	I had never thought about the fact that, that keeping them engaged, that is better for them. It keeps ‘em busy. And so I have really worked on, you know, trying to keep him engaged in what I’m doing. ‘Cause he’ll get up and he’ll say, you know, what’s the plan for the day? And I’ll say, well I need you in the house and this is what I need. . . . I start [laughs] I start off with one thing, I said now do this and when you get that done come back and I’ll give you something else to do. (P06)
Emphasis on caregiver well-being	[J]ust trying to get the support system group going and, not being so prideful when I do need help, to ask for help, instead of . . . trying to do everything myself. (P03)
**Gaps in the Cognitive Disorders Specialty Care Education Center of Excellence caregiver services**	
Provide additional information on dementia	You know, because I want to call it what it is. I don’t want to label something it’s not. . . . And . . . the stages. . . . and how you handle situation ‘cause that, you answer this lady question over here about her husband violent, you might be answering a question for me when that time come. (P02)
	So, you know, tell me anything that I . . . well, when I get to this final stage, you can expect such and such, you know. And what to, what you can do to make it easier on yourself . . . how you can continue to get the self-care that you need in order to be able to withstand that last, that final stage. (P09)
Provide additional support for individual caregiver challenges	I’m still . . . I mean, I was hoping it would help me, the obsession he has over the video tapes. That was something I would get some more concrete advice but even the doctors now they don’t . . . [laughs] I mean they don’t even really have any concrete things. Just handling it and . . . one thing that I’ve gotten most out of is just don’t take him where they have videos [laughs] so. But that’s hard to do sometimes. (P03)
Offer practical and logistical support	[As] a caregiver, all of a sudden, especially on a woman, lot of the roles that you didn’t play, your veteran usually would do a lot of the financial things, any kind of dealing in stocks, you know things like that. . . . If somebody . . . or even, legal stuff is a lot of things I wish they were call in, maybe, an elder attorney to help describe some of the ways that, things that you need to do to make it easier for you, especially if you need to have the patient’s signature ahead of time. Before they get to a part where they can’t sign things. . . . Or even to tell benefits that the VA could help you get, like that aide and attendance, some of the different things for that. (P01)
Improve course availability	I don’t get home until like 4 or 5 and then, by the time I get here, it wouldn’t be, it would be like 6 or 6:30 because of the traffic coming through. So it would have to be evening. Well then, I can’t do too much because he . . . his confusion comes in the evening. (P04)

a An in-depth, semistructured interview was conducted with each participant from November 2016 through February 2017 in private Veterans Affairs Health Care System offices or by telephone.

**Information about dementia** *.* Most caregivers (n = 10) appreciated dementia information that COE services provided. Information about dementia reinforced the notion that caregivers had no control over disease progression, reducing feelings of guilt or responsibility. Caregivers who enrolled in the formal psychoeducational programs (ie, Savvy Caregiver and Tele-Savvy) were pleased with how the curriculum offered organized information on dementia, including what is normal and future prognosis. These caregivers noted how program information was used to tailor their routines to the unique symptoms and circumstances of various dementia stages. Program instruction seemed less constructive for caregivers managing late-stage dementia, for which care can be more taxing and complicated. Instead, one caregiver sought palliative care information from the internet and other avenues. In addition, 4 caregivers expressed difficulty in comprehending dementia symptoms, disease stages, and other information used to describe the veteran’s illness.

**Social support.** Most caregivers who participated in the psychoeducational programs (n = 7) described the emotional benefits of interacting with other caregivers who were managing similar circumstances, noting a shared sense of empathy and encouragement. Caregivers were better able to “see what’s coming” in terms of disease progression through sharing anecdotes, which better prepared them for their role. Caregivers also described using personal experiences to assist others, which facilitated community building (ie, being “in here together”) and contrasted with instances when they repressed or shielded others from the difficulties of dementia caregiving. Additionally, caregivers discussed social support provided by COE clinic staff members who would purposefully reach out to caregivers to informally check in with them.

**Caregiver self-efficacy** *.* Seven caregivers noted increased self-efficacy, described as feeling more comfortable performing tasks typically ascribed to a caregiver, as a direct result of COE services. They recalled moments when they were better able to regulate their emotional responses to the veteran’s behaviors after participation in the psychoeducational programs. With improved self-efficacy, caregivers felt less overwhelmed by their circumstances and better equipped to address challenges through such strategies as prioritizing tasks. Improved self-efficacy was more apparent among caregivers who participated in the psychoeducational programs than among those who did not.

**Application of behavioral strategies.** Six of the 11 caregivers described moments of consciously altering their behavior to reflect information gained from the COE staff and support services in an effort to respond more competently to the veteran’s symptoms. These participants acknowledged using popular guidance from Savvy Caregiver materials, such as “Don’t just do something; stand there.” From this guidance, caregivers learned how to evaluate care recipient behavior and react supportively to each stage of the disease by using strategies such as meditation. Caregivers who had participated in the psychoeducational programs were more likely to be aware of appropriate behavioral strategies when caring for a patient with dementia and to adopt these practices into their routines.

**Emphasis on caregiver well-being.** Three caregivers noted difficulty in prioritizing personal needs because of the veteran’s illness and deteriorating condition. Participants were, therefore, more likely to neglect their own health and well-being, citing instances of failing to schedule needed medical appointments, being unable to exercise, and experiencing chronic stress and disrupted sleep. Caregivers described how COE support services encouraged continued self-care (ie, caring for the caregiver) and emphasized how caregivers may struggle to continue supporting the veteran if they do not prioritize caring for themselves. In some cases, increased caregiver focus on their own well-being after COE program participation resulted in sustained behavior changes, such as better sleep management. An emphasis on caregiver well-being also prompted 2 caregivers to seek greater external support from loved ones, something they previously avoided.

Caregivers identified 4 gaps and recommended future COE services: 1) provide additional information on dementia, 2) provide additional support for individual caregiver challenges, 3) offer practical and logistical support (eg, navigating VA benefits, financial tasks), and 4) improve availability of formal caregiver programs.

**Provide additional information on dementia.** Although caregivers appreciated dementia information provided through COE services, 5 caregivers expressed a desire to acquire more detailed information about the disease and disease management. In particular, caregivers sought a clearer picture of the disease’s expected pathology as a way to better manage their circumstances. Group interactions in COE programs also alerted caregivers to gaps in their dementia knowledge, and this prompted some to seek out additional dementia information and care that they may not have considered previously. Two caregivers were interested in information on end-of-life care, recognizing the importance of the topic but acknowledging that they felt overwhelmed about pursuing this discussion in detail.

**Provide additional support for individual caregiver challenges** *.* Seven caregivers emphasized a desire for support services that better addressed individual caregiver challenges. Three caregivers discussed alarming or frustrating behavior changes in the spouse that were likely manifesting because of dementia. One participant recalled her husband’s obsessive behavior, explaining how she would have appreciated targeted support on how to manage this unique symptom. Caregivers emphasized that some concerns, such as increased sex drive or dementia-related infidelity, may be too personal to discuss with family and asked that COE services offer time to problem-solve specific dementia quirks with the staff or as a group, especially because other caregivers may have experienced similar difficulties.

**Offer practical and logistical support** *.* As a facet of navigating the VA health system, 6 caregivers indicated a need for increased practical and logistical support. Practical and logistical support encompassed external resource linkages, clarification on VA benefits and eligibility, and guidance on practical and legal matters such as advanced directives, financial management, and long-term care arrangements. Caregivers who were not familiar with the VA appeared to be at a particular disadvantage when navigating eligibility. Other caregivers wanted instruction on practical caregiving tasks, such as a do-not-resuscitate order for the spouse while he is still able to consent. Some caregivers noted that their husbands had handled household activities such as paying bills and overseeing investments and requested supplementary guidance on these tasks.

**Improve Savvy Caregiver and Tele-Savvy course availability.** Three caregivers would have liked the formal courses to be extended either through advanced modules or by increasing the number of sessions offered. Two caregivers requested an extension of the program over longer time intervals, and one caregiver described how it was difficult to keep up with weekly readings and assignments while acting as a caregiver. To accommodate caregivers who work full-time or have other commitments, one participant requested these programs be held at alternative times instead of weekday daytime hours.

## Discussion

Our formative evaluation identified several components of COE caregiver services that were perceived as beneficial by participants. Caregivers responded positively to dementia information disseminated through COE services. This finding aligns with a previous meta-analysis of caregiver programs that found reductions in caregiver burden and emotional distress from interventions focused on increasing knowledge of the disease in an interactive format (18). To strengthen existing formal programs and resources that provide disease information, COE should consider expanding the current dementia curriculum for caregivers to provide supplementary details about the disease. With this updated curriculum, COE may address additional caregiver-requested content such as information that expands on introductory materials (eg, greater detail about disease stages), palliative care, and practical and logistical support. Some caregivers acknowledged challenges with comprehension of material on dementia topics. Research has shown that advanced health information may exacerbate caregiver distress if not presented in appropriate formats (19). COE should examine the health literacy levels of all dementia materials offered through caregiver services and refine materials as needed.

Consistent with previous research, caregivers attributed increased self-efficacy, behavior modification, and reduced feelings of caregiver burden to the Savvy Caregiver Program (13). Any expansion of COE services could consider methods to boost self-efficacy among caregivers of veterans beyond structured programs such as the Savvy Caregiver. Because self-efficacy is related to successfully completing tasks (20), caregiver self-efficacy may be improved through interactive mechanisms that encourage caregivers to share information about successfully managing their role. Additionally, novel alternatives such as home telehealth strategies would allow COE staff members to observe caregiving techniques used and offer constructive guidance (21).

Caregivers may neglect their needs or struggle to prioritize self-care while providing support to care recipients (22). Findings from our evaluation suggest that an emphasis on caregiver well-being functions as an impetus for behavior change, with caregivers citing moments when they chose to pursue self-care strategies after psychoeducational programs and COE staff guidance. COE may consider developing print and other materials on well-being practices for caregivers not already engaged in COE services to increase its reach to those who may struggle to prioritize their well-being.

Social support from interactions with other caregivers and COE clinic staff members was particularly well-received, echoing one meta-analysis that found caregiver support group participants demonstrated significant emotional gains (23). However, current COE caregiver support services are not structured to sustain long-term support groups because COE formal caregiver programs are time-limited. Although the COE is currently piloting remote delivery through the Tele-Savvy Program, its staff should continue to expand caregiver support to internet-based models. Asynchronous, informal online support groups may provide a feasible, cost-effective alternative to more traditional in-person formats, and recent work has demonstrated benefits from online support communities for dementia caregivers (24–26). Additionally, internet-based education and support interventions, as an alternative to in-person scheduled instruction, have also proven effective for dementia caregivers (27,28). Web-based delivery of information and support would address caregiver concerns about course availability, because they could access COE resources on their own schedule. COE may consider implementing any of these informal virtual strategies to enhance remote delivery of dementia information and to sustain social support.

Caregivers discussed a desire to engage with additional practical resources, similar to a previous study that found that only 19% of sampled caregivers were aware of how to access community services (29). COE could continue to offer and update a comprehensive resource list for caregivers that includes information on both health (eg, support groups) and practical resources (eg, legal counsel) to standardize and streamline referrals for external services in their local communities. In the future, COE may consider collaborating with other VA and external caregiver programs to expand services and to further address caregiver requests for more practical and logistical support.

Our study had several strengths. Because COE has documentation procedures for caregiver programs, such as rosters of psychoeducational program participants and clinical notes from caregiver interactions, we used well-maintained records to identify caregivers who had engaged in support services, which allowed us to capture multiple perspectives. Semistructured interviews provided caregivers with the appropriate conduit for more in-depth discussions of support services and the perceived effects these resources had on caregivers than quantitative surveys offer. In an attempt to reduce the likelihood of socially desirable responses, participants were informed that their feedback would not affect their benefits, and interviews were conducted by a third party not related to the provision of COE services.

Our formative evaluation also had some limitations. Because our analysis was qualitative and the sample size was small, findings are not generalizable to other caregiver populations or clinic environments outside the Atlanta VAHCS. However, the purpose of qualitative data is to provide insight, not generalizability, and the experiences of this sample aligned with published research on caregiver burden. Findings are also restricted to caregivers who engaged in caregiver support resources and who were interested in offering their perspectives on the program.

COE is in the process of collecting complementary survey data before and after program participation to assess how factors related to caregiver burden, such as stress and depression, are influenced by COE support services and to bolster these qualitative findings. Future work should seek to explore the perspectives of caregivers who are less engaged in these services to improve reach. Future efforts may also incorporate staff perspectives on caregiver support services, a useful vantage point when considering program feasibility and sustainability. Any expansion or translation of COE caregiver services should ultimately undergo a full program evaluation.

Caregiver support services piloted by COE are well-regarded among dementia caregivers who use these resources to better manage the challenging circumstances and responsibilities of their role. Although caregivers noted some important limitations of these services that should be considered in the future, COE support services were found to be beneficial to caregivers through such mechanisms as increased knowledge of dementia, greater social support, increased self-efficacy to care for the veteran, behavior modification, and an emphasis on caregiver well-being. Findings from our formative evaluation support the continuation and expansion of these COE programs and demonstrate the usefulness of providing caregiver services through VA facilities to improve the care and quality of life of veterans living with dementia and their caregivers.

## References

[1] Hurd MD , Martorell P , Delavande A , Mullen KJ , Langa KM . Monetary costs of dementia in the United States. N Engl J Med 2013;368(14):1326–34. 10.1056/NEJMsa1204629 23550670 PMC3959992

[2] Plassman BL , Langa KM , Fisher GG , Heeringa SG , Weir DR , Ofstedal MB , Prevalence of dementia in the United States: the aging, demographics, and memory study. Neuroepidemiology 2007;29(1-2):125–32. 10.1159/000109998 17975326 PMC2705925

[3] Prince M , Bryce R , Albanese E , Wimo A , Ribeiro W , Ferri CP . The global prevalence of dementia: a systematic review and metaanalysis. Alzheimers Dement 2013;9(1):63–75.e2. 10.1016/j.jalz.2012.11.007 23305823

[4] National Alliance for Caregiving and the AARP Public Policy Institute. Caregiving in the U.S.; 2015. http://www.caregiving.org/wp-content/uploads/2015/05/ 2015_CaregivingintheUS_Final-Report-June-4_WEB.pdf. Accessed January 30, 2018.

[5] Wolff JL , Spillman BC , Freedman VA , Kasper JD . A national profile of family and unpaid caregivers who assist older adults with health care activities. JAMA Intern Med 2016;176(3):372–9. 10.1001/jamainternmed.2015.7664 26882031 PMC4802361

[6] Moon H , Rote S , Beaty JA . Caregiving setting and Baby Boomer caregiver stress processes: findings from the National Study of Caregiving (NSOC). Geriatr Nurs 2017;38(1):57–62. 10.1016/j.gerinurse.2016.07.006 27520990

[7] Schulz R , Sherwood PR . Physical and mental health effects of family caregiving. Am J Nurs 2008;108(9, Suppl):23–7, quiz 27. 10.1097/01.NAJ.0000336406.45248.4c 18797217 PMC2791523

[8] Zarit SH , Todd PA , Zarit JM . Subjective burden of husbands and wives as caregivers: a longitudinal study. Gerontologist 1986;26(3):260–6. 10.1093/geront/26.3.260 3721233

[9] Nichols LO , Martindale-Adams J , Zhu CW , Kaplan EK , Zuber JK , Waters TM . Impact of the REACH II and REACH VA dementia caregiver interventions on healthcare costs. J Am Geriatr Soc 2017;65(5):931–6. 10.1111/jgs.14716 28295134 PMC5435565

[10] Mittelman MS , Ferris SH , Shulman E , Steinberg G , Ambinder A , Mackell JA , A comprehensive support program: effect on depression in spouse-caregivers of AD patients. Gerontologist 1995;35(6):792–802. 10.1093/geront/35.6.792 8557206

[11] Bass DM , Judge KS , Snow AL , Wilson NL , Morgan R , Looman WJ , Caregiver outcomes of partners in dementia care: effect of a care coordination program for veterans with dementia and their family members and friends. J Am Geriatr Soc 2013;61(8):1377–86. 10.1111/jgs.12362 23869899

[12] Gitlin LN , Jacobs M , Earland TV . Translation of a dementia caregiver intervention for delivery in homecare as a reimbursable Medicare service: outcomes and lessons learned. Gerontologist 2010;50(6):847–54. 10.1093/geront/gnq057 20660473 PMC2982211

[13] Hepburn KW , Lewis M , Sherman CW , Tornatore J . The Savvy Caregiver program: developing and testing a transportable dementia family caregiver training program. Gerontologist 2003;43(6):908–15. 10.1093/geront/43.6.908 14704391

[14] Lazarus RS , Folkman S . Transactional theory and research on emotions and coping. Eur J Pers 1987;1(3):141–69. 10.1002/per.2410010304

[15] Griffiths PC , Whitney MK , Kovaleva M , Hepburn K . Development and implementation of Tele-Savvy for dementia caregivers: a Department of Veterans Affairs clinical demonstration project. Gerontologist 2016;56(1):145–54. 10.1093/geront/gnv123 26566806

[16] Kovaleva MA , Bilsborough E , Griffiths PC , Nocera J , Higgins M , Epps F , Testing Tele-Savvy: protocol for a randomized controlled trial. Res Nurs Health 2018;41(2):107–20. 10.1002/nur.21859 29399825 PMC5945279

[17] Corbin J , Strauss A . Grounded theory research: procedures, canons, and evaluative criteria. Qual Sociol 1990;13(1):3–21. 10.1007/BF00988593

[18] Pinquart M , Sörensen S . Helping caregivers of persons with dementia: which interventions work and how large are their effects? Int Psychogeriatr 2006;18(4):577–95. 10.1017/S1041610206003462 16686964

[19] Proctor R , Martin C , Hewison J . When a little knowledge is a dangerous thing . . . : a study of carers’ knowledge about dementia, preferred coping style and psychological distress. Int J Geriatr Psychiatry 2002;17(12):1133–9. 10.1002/gps.762 12461762

[20] Bandura A . Self-efficacy: toward a unifying theory of behavioral change. Psychol Rev 1977;84(2):191–215. 10.1037/0033-295X.84.2.191 847061

[21] Williams K , Arthur A , Niedens M , Moushey L , Hutfles L . In-home monitoring support for dementia caregivers: a feasibility study. Clin Nurs Res 2013;22(2):139–50. 10.1177/1054773812460545 22997349 PMC3633651

[22] Lilly MB , Robinson CA , Holtzman S , Bottorff JL . Can we move beyond burden and burnout to support the health and wellness of family caregivers to persons with dementia? Evidence from British Columbia, Canada. Health Soc Care Community 2012;20(1):103–12. 10.1111/j.1365-2524.2011.01025.x 21851447

[23] Chien LY , Chu H , Guo JL , Liao YM , Chang LI , Chen CH , Caregiver support groups in patients with dementia: a meta-analysis. Int J Geriatr Psychiatry 2011;26(10):1089–98. 10.1002/gps.2660 21308785

[24] O’Connor MF , Arizmendi BJ , Kaszniak AW . Virtually supportive: a feasibility pilot study of an online support group for dementia caregivers in a 3D virtual environment. J Aging Stud 2014;30:87–93. 10.1016/j.jaging.2014.03.001 24984911 PMC4668714

[25] McKechnie V , Barker C , Stott J . The effectiveness of an Internet support forum for carers of people with dementia: a pre-post cohort study. J Med Internet Res 2014;16(2):e68. 10.2196/jmir.3166 24583789 PMC3961748

[26] Marziali E , Garcia LJ . Dementia caregivers’ responses to 2 Internet-based intervention programs. Am J Alzheimers Dis Other Demen 2011;26(1):36–43. 10.1177/1533317510387586 21282276 PMC10845422

[27] Boots LM , de Vugt ME , van Knippenberg RJ , Kempen GI , Verhey FR . A systematic review of Internet-based supportive interventions for caregivers of patients with dementia. Int J Geriatr Psychiatry 2014;29(4):331–44. 10.1002/gps.4016 23963684

[28] Beauchamp N , Irvine AB , Seeley J , Johnson B . Worksite-based internet multimedia program for family caregivers of persons with dementia. Gerontologist 2005;45(6):793–801. 10.1093/geront/45.6.793 16326661

[29] Jennings LA , Reuben DB , Evertson LC , Serrano KS , Ercoli L , Grill J , Unmet needs of caregivers of individuals referred to a dementia care program. J Am Geriatr Soc 2015;63(2):282–9. 10.1111/jgs.13251 25688604 PMC4332558

